# Supplementary material for: Experimental realisation of multi-qubit gates using electron paramagnetic resonance
Source: Nat Commun. 2023 Nov 2;14:7029. doi: 10.1038/s41467-023-42169-7 (PMC10622571; doi:10.1038/s41467-023-42169-7)
Supplement: Supplementary file 1 — Supplementary Information [file 41467_2023_42169_MOESM1_ESM.pdf]

Supplementary Information

Experimental Realisation of Multi-Qubit Gates Using Electron Paramagnetic Resonance – Supplementary Information

Edmund J. Little[1], Jacob Mrozek[2], Ciarán J. Rogers[1], Junjie Liu[2], Eric J. L. McInnes[1], Alice M. Bowen[1]*, Arzhang Ardavan[2]*, Richard E. P. Winpenny[1]*

[1]Photon Science Institute and School of Chemistry, The University of Manchester, Oxford Road, Manchester, M13 9PL, United Kingdom
[2]Clarendon Laboratory, University of Oxford, Parks Road, Oxford, OX1 3PU, United Kingdom

CONTENTS

SUPPLEMENTARY NOTE 1 SYNTHESIS AND CHARACTERISATION 2

SUPPLEMENTARY NOTE 2 THEORY 4

SUPPLEMENTARY NOTE 3 RESULTS 8

SUPPLEMENTARY NOTE 4 PRODUCT OPERATOR CALCULATIONS 17

SUPPLEMENTARY NOTE 5 NUMERICAL SIMULATIONS 21

SUPPLEMENTARY REFERENCES 22

Supplementary Note 1 Synthesis and Characterisation

To a solution of 4-iodobenzoic acid (0.5 g, 2.02 mmol) and 4-amino-TEMPO (0.41 g, 2.39 mmol) in anhydrous DMF (7 mL) was added EDCI.HCl (0.48 g, 2.5 mmol) and HOBt (0.48 g, 3.5 mmol). The reaction mixture was stirred at room temperature under an atmosphere of N_2 for 12 h before concentration in vacuo. The crude product was purified by chromatography on silica gel (EtOAc/Hexanes:4/6) to give IBzTEMPO as orange needle-like crystals (0.56 g, 70%). ESI m/z 424 [$M+Na^+$], calculated 424.07 for [$M+Na^+$]: $C_{16}H_{22}N_2O_2I$.

A 100 mL dry round bottomed flask was vacuumed and filled with nitrogen gas to which IBzTEMPO (0.1 g, 0.25 mmol), 1,4-diethynylbenzene (0.015 g, 0.12 mmol), Palladium-tetrakis(triphenylphosphine) (12.5% mol, 0.020 g, 0.015 mmol), copper(I) iodide (0.020 g) and dry THF (15 mL) was added. Dry triethylamine (NEt3) (5 mL) was subsequently added and the reaction mixture allowed to stir for 8 h at 50°C, and then room temperature for 40 h. The crude product was filtered through a layer of celite and the solution concentrated in vacuo before purification by chromatography on silica gel ($CHCl_3$/EtOAc:8/2) to give compound **1** as a pale yellow powder (0.056 g, 69%). ESI m/z 671.3608 [$M-H^+$], calculated 671.3603 for [$M-H^+$]: $C_{42}H_{47}O_4N_4$. Elemental Analysis: calculated (found) for $C_{42}H_{47}O_4N_4$: C, 74.97 (73.5); H, 7.19 (7.23); N, 8.33 (7.98). Procedure adapted from reference[1].

Experimental Electron Paramagnetic Resonance Spectroscopy

All pulse EPR data were collected using a Bruker E580 spectrometer equipped with a SpinJet Arbitrary Waveform Generator and MD5 resonator at 50K on a 0.2 mM solution of compound **1** made up in a solution of equal parts toluene and chloroform.

Echo detected field-swept spectra were collected using a Hahn-echo sequence $t_{90^\circ} - \tau - t_{180^\circ}$, with t_{90° = 20 ns, t_{180° = 40 ns, and τ = 400 ns. All frequency-static experiments were performed at 342.9 mT, except the 2P-ESEEM and amplifier characterisation which were performed at 342.7 mT. Transient nutations for hardware characterisation used the pulse sequence $t - T - t_{90^\circ} - \tau - t_{180^\circ}$

with $t_{90°}$ = 20 ns, $t_{180°}$ = 40 ns, τ = 800 ns, T = 25 µs, and an initial t = 0 ns. 2P-ESEEM data were collected using the sequence $t_{90°} - \tau - t_{180°}$ with $t_{90°}$ = 100 ns, $t_{180°}$ = 200 ns, and an initial τ of 1 µs. DFHE-decay data were collected using the sequence $t_{90°} - \tau - t_{180°}$, $t_{90°}$ = 80 ns, $t_{180°}$ = 80 ns and an initial τ of 0 ns. 3P-DEER data used the detection sequence $t_{90°} - \tau - t_{180°}$, with $t_{90°}$ = 80 ns, $t_{180°}$ = 80 ns and τ = 1.5 µs, using an 80 ns pump pulse. 5P-DFHE-DEERATom data used the detection sequence $t_{90°} - \tau - t_{180°} - \tau - t_{\mathrm{var}} - T - t_{180°} - T$ with $t_{90°}$ = 80 ns, $t_{180°}$ = 80 ns, τ = 250 ns, and T = 1.5 µs and with an 80 ns pump pulse, and t_{var} = 80 ns when active. All delay times are defined to be between the start of consecutive pulses.

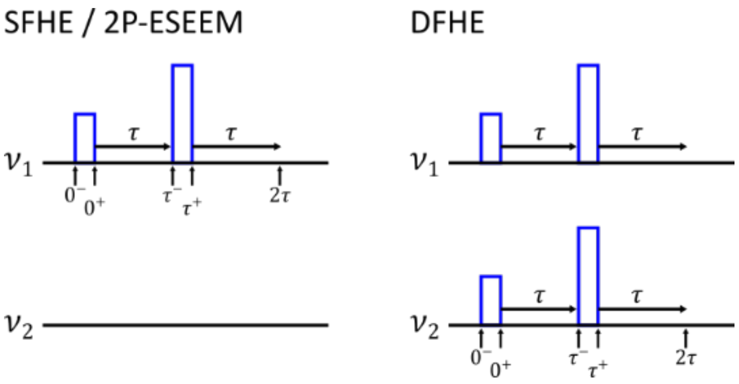

Supplementary Figure 1: Comparison of the Single-Frequency Hahn Echo (SFHE) and Dual Frequency Hahn Echo (DFHE) pulse sequences, with 90° pulses shown at half the height of 180° pulses. These can be converted into echo decay experiments by incrementing the inter-pulse delay τ, in which case the SFHE is equivalent to the 2P-Electron Spin Echo Envelope Modulation (2P-ESEEM) experiment. The labels of the frequencies are arbitrary and so ν_1 can be either ν_{S} or ν_{I}, in which case ν_2 would be ν_{I} or ν_{S}, respectively.

Supplementary Table 1: The operators which lead to oscillations in the echo intensity of a 'pump-refocus' block in the real (in-phase, I) and imaginary (quadrature, Q) channels, assuming that the refocussing pulse is in $+x$.

Channel	Phase	Detection ν_1	Detection ν_2
Q	cos	$\hat{S}_x$	$\hat{I}_x$
I	cos	$\hat{S}_y$	$\hat{I}_y$
I	sin	$2\hat{S}_x\hat{I}_z$	$2\hat{S}_z\hat{I}_x$
Q	sin	$2\hat{S}_y\hat{I}_z$	$2\hat{S}_z\hat{I}_y$

Supplementary Note 2 Theory

Supplementary Note 2.1 Relation of time traces to expectation values

The most general description of a dipolar time trace is:

$$V(t) = F(t)B(t) + c \quad (1)$$

where $F(t)$ is the form factor, $B(t)$ is the background function, and c is the acquisition offset.[2] The acquisition offset is caused by imperfect calibration of the detection voltages and is generally cancelled out by a phase cycle. In the absence of phase cycling this represents a constant proportional to the number of scans. The background function is caused by low frequency inter-molecular inter-spin interactions, which give rise to low frequency oscillations in the time trace and in homogeneous samples can be modelled as an exponential decay, $B(t) = e^{-kt}$.[3] The decay rate k depends on (amongst other things) the spin concentration in the sample and the excitation fraction of the pump pulse. The form factor contains all intra-molecular information, and can be expressed as:

$$F(t) = F_0 - \lambda S_0 + \lambda S^{\mathrm{C}}(t) + \mu S^{\mathrm{S}}(t) \quad (2)$$

where F_0 is the unmodulated echo intensity, S_0 is the amplitude of the modulations, $S^{\mathrm{C}}(t)$ is the cosine modulation function, $S^{\mathrm{S}}(t)$ is the sine modulation function, λ is the depth of the cosine modulation, and μ is the depth of the sine modulation. The modulation functions can be given as:

$$S^{\mathrm{C}}(t) = \frac{S_0}{n}\sum_{i}^{n}\cos(\omega_{\mathrm{AB}}^{i}t)\,;\; S^{\mathrm{S}}(t) = \frac{S_0}{n}\sum_{i}^{n}\sin(\omega_{\mathrm{AB}}^{i}t) \quad (3)$$

where n is the subpopulation of molecules in the sample in which one spin is detected and the other is inverted by the pump pulse, and ω_{AB}^{i} is the coupling frequency of the spin pair. If the anisotropy of the spin systems can be neglected ($g_x \approx g_y \approx g_z$ for both spins) and the high-field limit applies ($|\omega_{\mathrm{A}} - \omega_{\mathrm{B}}| \ll \omega_{\mathrm{AB}}$) then the inter-spin interaction is given by:

$$\omega_{\mathrm{AB}}^{i} = \frac{\mu_0 g_{\mathrm{A}}^{i} g_{\mathrm{B}}^{i} \mu_{\mathrm{B}}^{2}}{4\pi\hbar} \frac{1}{{r_{\mathrm{AB}}^{i}}^{3}} \left(3\cos^2\left(\theta_{\mathrm{AB}}^{i}\right) - 1\right) + J_{\mathrm{AB}}^{i} \qquad 4$$

where μ_0 is the vacuum permeability, μ_{B} is the Bohr magneton, $\hbar$ is the reduced Planck constant, g_{A}^{i} and g_{B}^{i} are the g-values of the two spins, r_{AB}^{i} is the inter-spin distance, θ_{AB}^{i} is the angle between the inter-spin vector and the external magnetic field, and J_{AB}^{i} is the super-exchange (through-bond) interaction between the two spins.[4]

The remaining parameters in the form factor relate to the state of the system before the application of the detection sequence. The unmodulated echo intensity is $F_0 = \alpha\langle\hat{O}_{\mathrm{in}}\rangle_{\mathrm{d}}$, where $\hat{O}_{\mathrm{in}}$ is the relevant in-phase spin operator for the experiment, the subscript d denotes the expectation value over the sub-population of spins resonant with the detection frequency, and α is a proportionality constant. Similarly, the modulation depths can be expressed $\lambda = \beta\langle\hat{O}_{\mathrm{in}}\rangle_{\mathrm{d}\wedge\mathrm{p}}$ and $\mu = \beta\langle\hat{O}_{\mathrm{anti}}\rangle_{\mathrm{d}\wedge\mathrm{p}}$, where $\hat{O}_{\mathrm{anti}}$ is the relevant anti-phase operator, the subscript $\mathrm{d} \wedge \mathrm{p}$ denotes the expectation value is taken over the sub-population of spins resonant with the detection frequency whose partner spins are inverted by the pump pulse, and β is a proportionality constant smaller than α as it accounts for partial inversion of partner spins.

Supplementary Note 2.2 Pseudo-fidelity

State fidelity metrics aim to provide a numerical value for the similarity between two quantum states, such as $\mathcal{F}(\hat{\rho}_1, \hat{\rho}_2) = \left[\mathrm{tr}\left(\sqrt{\sqrt{\hat{\rho}_1}\hat{\rho}_2\sqrt{\hat{\rho}_1}}\right)\right]^2$.[5] For magnetic resonance measurements in the high temperature limit, the both states will be dominated by the identity element, and so their fidelity by this metric will be approximately equal to one. A more meaningful metric must compare their deviation density matrices, which we accomplish by treating the expectation values of the non-identity Cartesian product operators as vectors in the generalised Bloch sphere. In this representation the similarity between two states can be thought of as the angle between them, and we use the cosine of this angle to define the pseudo-fidelity:

$$\tilde{\mathcal{F}}(\hat{\rho}_1,\hat{\rho}_2) = \frac{d-1}{2d}\cos\angle(\hat{\rho}_1,\hat{\rho}_2) + \frac{d+1}{2d}$$

where d is the Hilbert dimension, and:

$$\begin{aligned}\cos\angle(\hat{\rho}_1,\hat{\rho}_2) &= \vec{\rho}_1 \cdot \vec{\rho}_2 \\ &= \sum_i^n \alpha_i \beta_i \\ &= \mathrm{tr}(\hat{\rho}_1\hat{\rho}_2) - \frac{1}{d}\end{aligned}$$

where $\vec{\rho}$ is the vector representation of $\hat{\rho}$, and α and β are the expectation values of the basis operators in $\hat{\rho}_1$ and $\hat{\rho}_2$, respectively (i.e. the elements of $\vec{\rho}_1$ and $\vec{\rho}_2$). Note that this is not a generally acceptable metric as it does not fulfil many requirements of true fidelity metrics, however, $\tilde{\mathcal{F}}(\hat{\rho}_1,\hat{\rho}_1) = 1$, $\tilde{\mathcal{F}}(\hat{\rho}_1,\hat{\rho}_2) = \tilde{\mathcal{F}}(\hat{\rho}_2,\hat{\rho}_1)$, and $\tilde{\mathcal{F}}(\hat{\rho}_1,\hat{\rho}_2) = \tilde{\mathcal{F}}(U\hat{\rho}_1U^\dagger, U\hat{\rho}_2U^\dagger)$ where U is a unitary operation. Further, if $\hat{\rho}_1$ and $\hat{\rho}_2$ are both pure $\tilde{\mathcal{F}}(\hat{\rho}_1,\hat{\rho}_2) = \mathcal{F}(\hat{\rho}_1,\hat{\rho}_2)$. If the absolute magnitude of the observables cannot be determined, it is common to normalise the vectors, resulting in a normalised pseudo-fidelity:

$$\tilde{\mathcal{F}}^{\mathrm{N}}(\hat{\rho}_1,\hat{\rho}_2) = \frac{d-1}{2d}\cos\angle^{\mathrm{N}}(\hat{\rho}_1,\hat{\rho}_2) + \frac{d+1}{2d}$$

where:

$$\begin{aligned}\cos\angle^{\mathrm{N}} &= \frac{\vec{\rho}_1}{|\vec{\rho}_1|} \cdot \frac{\vec{\rho}_2}{|\vec{\rho}_1|} \\ &= \frac{\sum_i^n \alpha_i \beta_i}{\sqrt{\sum_i^n \alpha_i^2 \sum_i^n \beta_i^2}} \\ &= \frac{\mathrm{tr}(\hat{\rho}_1\hat{\rho}_2) - \frac{1}{d}}{\sqrt{\left[\mathrm{tr}(\hat{\rho}_1^2) - \frac{1}{d}\right]\left[\mathrm{tr}(\hat{\rho}_2^2) - \frac{1}{d}\right]}}\end{aligned}$$

It should be noted that the fitted experimental state was normalised by the magnitude of the calculated expectation values because the experimental starting state is unknown. This means that the measurement is insensitive to an overall loss in magnetisation, and therefore this measurement of this pseudo-fidelity is a normalised pseudo-fidelity and an upper-bound (see further discussion later in the 'Relation to experiment' section in the main text).

Supplementary Note 3 Results

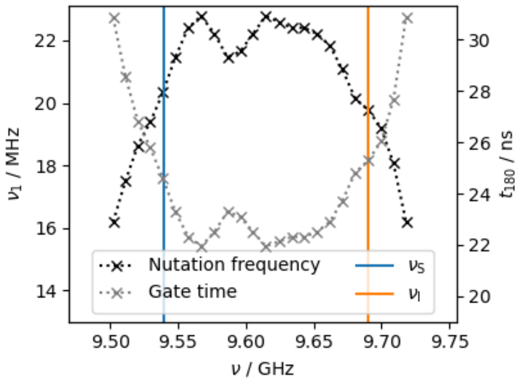

Supplementary Figure 2: Dependence of nutation frequency (black) and single qubit gate times (grey) on the frequency of the applied microwave. Dashed lines have been added between datapoints as a visual aid. The solid vertical lines denote at ν_S (9.54 GHz, blue) and ν_I (9.69 GHz, orange). Experimental details given in the supplementary information, section 2.

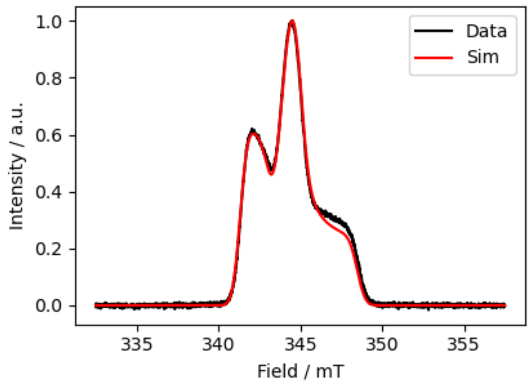

Supplementary Figure 3: Echo detected field swept spectrum of compound **1** at 9.665 GHz (black) and corresponding simulation (red). Simulation was performed with $[g_x, g_y, g_z]$ = [2.0093, 2.0054, 2.0020], hyperfine coupling to a single 14N nucleus A_x = A_y = 24.5 MHz, A_z = 98.8 MHz, and anisotropic line broadening $[H_x, H_y, H_z]$ = [27.2, 34.5, 25.1] MHz. Simulations were performed using the Matlab toolbox Easyspin.[6]

Supplementary Note 3.1 Data Handling

Transient nutation data for hardware characterisation were prepared for Fourier transform by subtracting a linear baseline, applying a Hamming window, and zero-filling to 2^{14} points. The complex Fourier transform was taken, and the nutation frequency determined to be that with the largest absolute intensity. The resonator profile should be approximately independent of the pulse power, and so to avoid proton ESEEM oscillations the amplitude of the nutation pulse was set to 10%, and the resulting nutation frequencies scaled to match an equivalent profile with 100% amplitude.

A similar process was performed to Fourier transform dipolar data, with data being rephased to minimise the imaginary component over the last three quarters of the trace before removing a mono-exponential background (see Supplementary Section 0). A Hamming window was applied, and the data were zero-filled to 2^{10} points before taking the real part of the complex Fourier transform. The rephased data for the traces shown in Figure 5 are shown in Supplementary Figure 4 prior to background correction and normalisation.

To determine the phase memory time, 2P-ESEEM data were fitted to a stretched exponential function $y = Ae^{-\left(\frac{2\tau}{T_{\mathrm{m}}}\right)^{\beta}} + c$. The fitted values were T_{m} = 6.89 μs and β = 1.54.

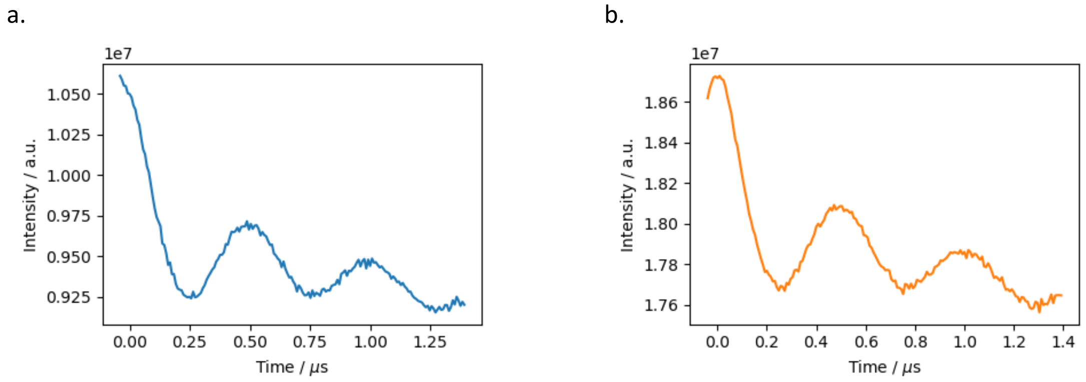

Supplementary Figure 4: Experimental 3P-DEER traces detecting at a. 9.54 GHz and b. 9.69 GHz.

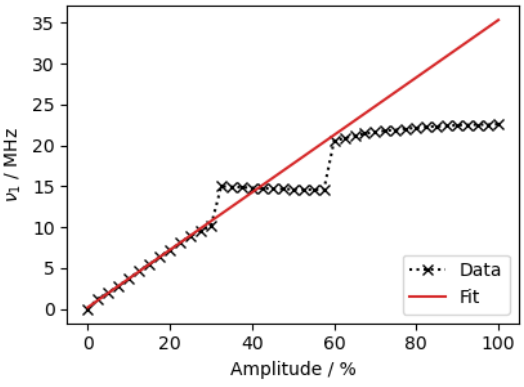

Supplementary Figure 5: Nutation frequency as a function of pulse amplitude (black), recorded on the signal maximum at 9.615 GHz and 343 mT. The linear fit (red) was performed over the region 0% to 25% amplitude. The region between 30% and 60% does not represent a true nutation frequency, instead corresponding to the detection of the proton nuclear Larmor frequency by weak hyperfine coupling of the electron spin.

Supplementary Note 3.2 DEERATom Data Analysis

The thirty-six DEERATom time traces were fitted simultaneously as raw data, with the background decay parameters fixed from the associated DEER data and offsets fitted independently for each trace. To account for partial excitation of the magnetisation by the filter pulses, the form factor of each trace was treated as weighted sum over filtered, partially filtered, and unfiltered magnetisation:

$$F(t) = P_{\mathrm{flip}}^2 F^{\mathrm{F_s F_I}}(t) + P_{\mathrm{flip}} Q_{\mathrm{flip}} [F^{\mathrm{F_s 1_I}}(t) + F^{\mathrm{1_s F_I}}(t)] + Q_{\mathrm{flip}}^2 F^{\mathrm{1_s 1_I}}(t) \qquad 5$$

where P_{flip} is the probability that the filter pulse is active, $Q_{\mathrm{flip}} = 1 - P_{\mathrm{flip}}$ is the probability that the filter pulse is inactive, and the superscripts F and 1 denote whether the filter pulse at the specified frequency is active or not. For each component of the weighted sum, the form factor was calculated by applying the appropriate combination of filter pulses to a density matrix of non-coupled spins, $\hat{\rho}^{\mathrm{nc}}$, to determine F_0, and to a separate coupled density matrix, $\hat{\rho}^{\mathrm{c}}$, to determine λ and μ. All expectation values were calculated from analytical expressions (i.e. rotations of triads) rather than by matrix exponentiation.[8]

The cosine modulation functions $S^{\mathrm{C}}(t)$ were the experimental DEER trace recorded at the corresponding frequency. The sine modulation functions $S^{\mathrm{S}}(t)$ were calculated from these by discrete cosine transform followed by inverse sine transform after appropriately shifting the frequency axis to account for the difference in indexing. Modulation functions were scaled such that $S_0 = \lambda^{\mathrm{D}} F_0$, where λ^{D} denotes the modulation depth of the corresponding DEER data, and thus the fitted values of λ and μ are independent of modulation depth and signal intensity and can be compared between all experiments.

To avoid over-weighting more intense traces in the global fit, the residuals of each point were weighted by division by its experimental value. As such the residuals are fractional deviations, i.e. for a given point i the residual is given by $R_i = \left|\frac{(\hat{y}_i - y_i)}{y_i}\right|$. The first 40 ns of each trace were ignored during the fit to minimise the impact of distortions due to the pump and off-detection filter pulses

overlapping in time. The resulting fitted coupled density matrix was then rotated about z to maximise its pseudo-fidelity to a reference density matrix of an ideal entangling gate, $\hat{\rho}^{\mathrm{ref}} = \hat{S}_x\hat{I}_z + \hat{S}_z\hat{I}_x$, accounting for errors in pulse and detection phases.

The fit procedure was repeated for a range of filter pulse flip probabilities, ranging from 0.01 to 1.00 in steps of 0.01. While the highest pseudo-fidelity of 0.790 was found when P_{flip} = 0.45, while the lowest cost occurred at P_{flip} = 0.42, for which the pseudo-fidelity was marginally lower at 0.789 (Supplementary Figure 6). All fits shown correspond to the lowest cost solution. Supplementary Figures 7, 8 and 9 show the normalised, background corrected time traces and corresponding fits for all pairs of operations.

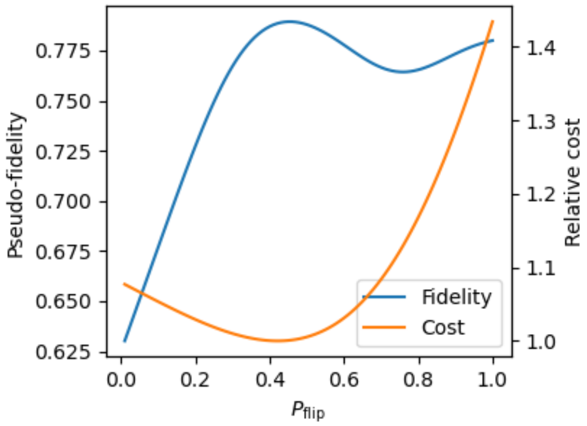

Supplementary Figure 6: The cost and pseudo-fidelity of the fits performed for a range of filter pulse flip probabilities.

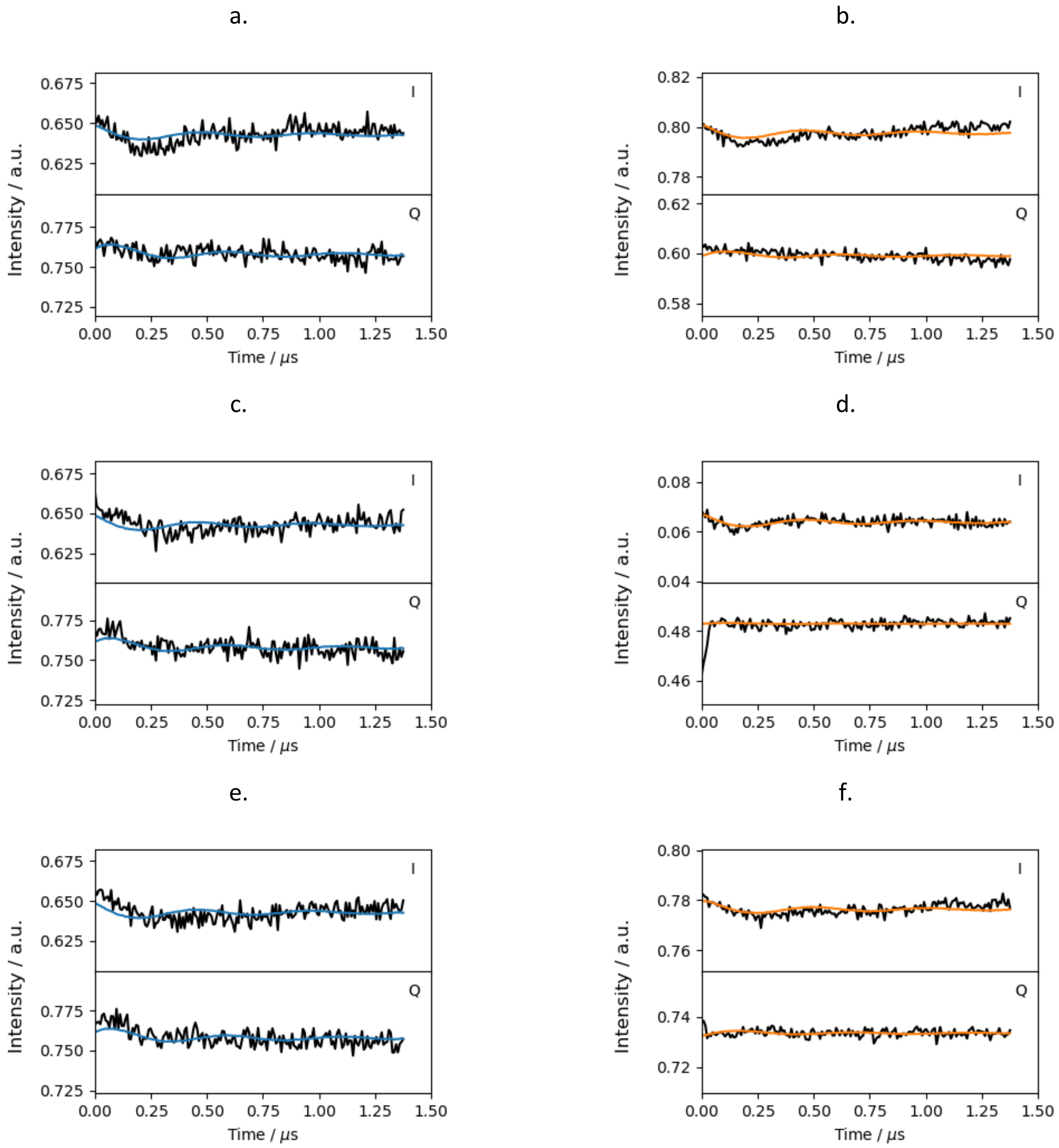

Supplementary Figure 7: Experimental DEERATom time traces (black) and corresponding fits (blue and orange) recorded with the pairs of operations a. 1_S1_I detected at ν_S, b. 1_S1_I detected at ν_I, c. 1_SX_I detected at ν_S, d. 1_SX_I detected at ν_I, e. 1_SY_I detected at ν_S, and f. 1_SY_I detected at ν_I. All data have been background corrected and normalised, with the real component denoted as I and the imaginary part as Q.

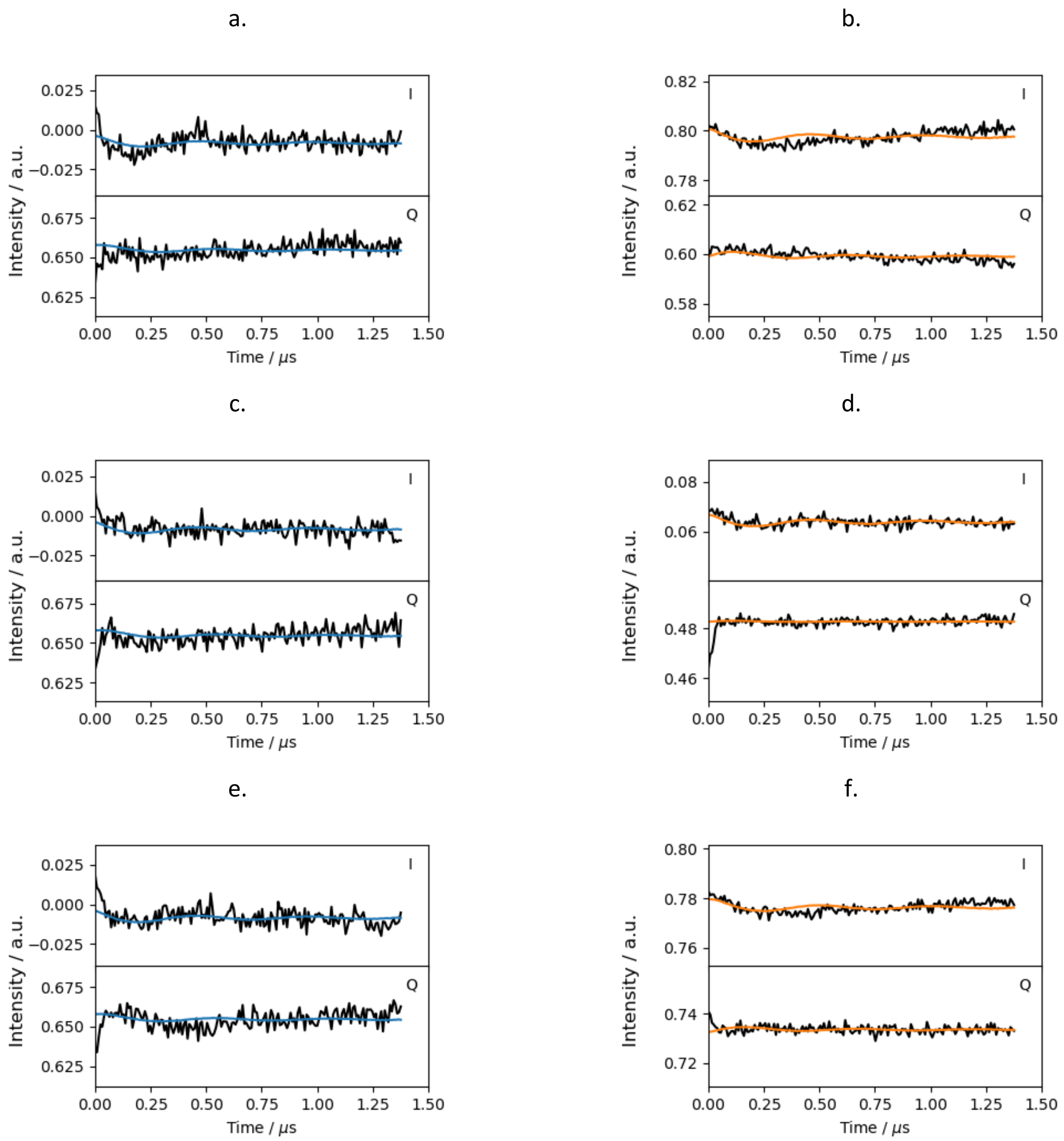

Supplementary Figure 8: Experimental DEERATom time traces (black) and corresponding fits (blue and orange) recorded with the pairs of operations a. $X_S 1_I$ detected at ν_S, b. $X_S 1_I$ detected at ν_I, c. $X_S X_I$ detected at ν_S, d. $X_S X_I$ detected at ν_I, e. $X_S Y_I$ detected at ν_S, and f. $X_S Y_I$ detected at ν_I. All data have been background corrected and normalised, with the real component denoted as I and the imaginary part as Q.

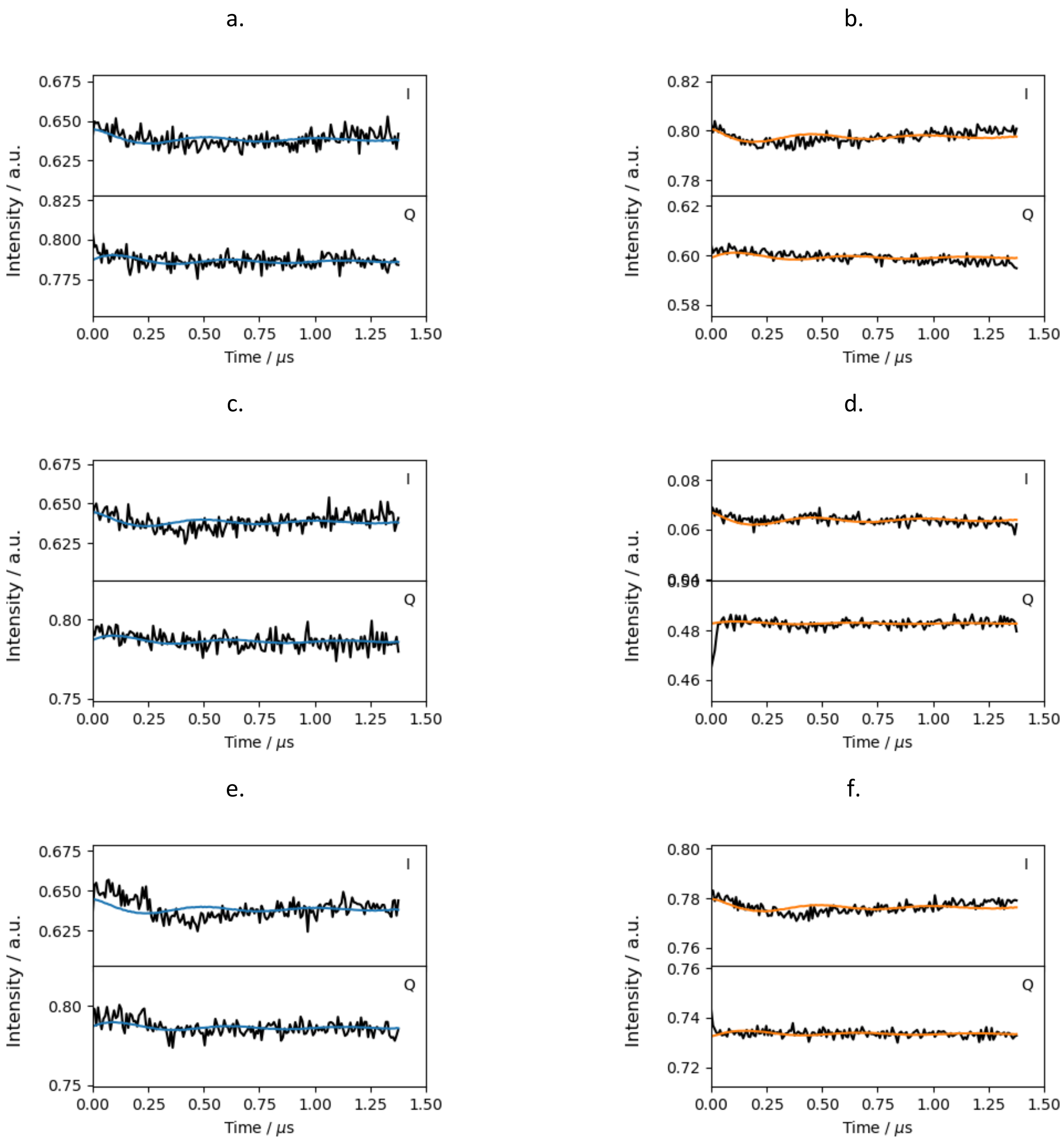

Supplementary Figure 9: Experimental DEERATom time traces (black) and corresponding fits (blue and orange) recorded with the pairs of operations a. $\mathrm{Y_S 1_I}$ detected at ν_S, b. $\mathrm{Y_S 1_I}$ detected at ν_I, c. $\mathrm{Y_S X_I}$ detected at ν_S, d. $\mathrm{Y_S X_I}$ detected at ν_I, e. $\mathrm{Y_S Y_I}$ detected at ν_S, and f. $\mathrm{Y_S Y_I}$ detected at ν_I. All data have been background corrected and normalised, with the real component denoted as I and the imaginary part as Q.

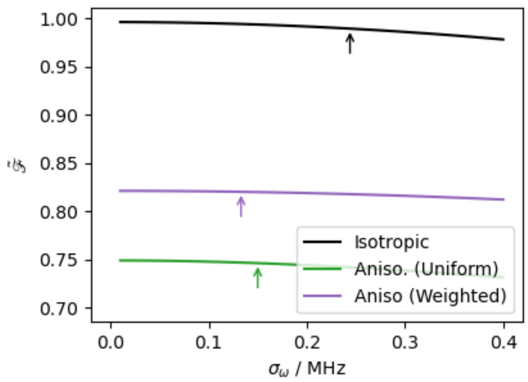

Supplementary Figure 10: Simulated pseudo-fidelity of the DFHE entangling gate as a function of standard deviation for normal distributions of inter-spin coupling. For the isotropic coupling model, $\omega \equiv \omega_{SI}$, and for the anisotropic models $\omega \equiv \omega_{\perp}$. Standard deviations corresponding to our experimental setup are marked with arrows, determined by fitting DEER traces to the respective models (Figure S5).

Supplementary Note 3.3 DEER Data Analysis

To determine physically reasonable distributions of parameters, the DEER data were fitted using DEERLab to the relevant kernels, using a gaussian distribution over the variable parameter (either ω_{SI} for the exchange model or $\omega_{\perp}$ for dipolar models) with centre μ and standard deviation σ.[7] The initial echo amplitude V_0, modulation depth λ, reference time t_0, and background spin concentration c_S were also fitted. Optimal values are shown in Supplementary Table 2 and the corresponding data and fits in Supplementary Figure 11.

a. b.

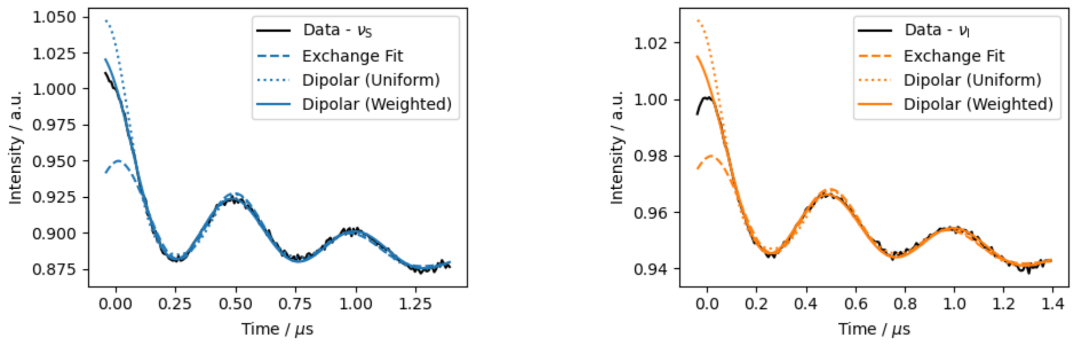

Supplementary Figure 11: Experimental 3P-DEER time traces (black) and corresponding fits (blue and orange) detected at a. ν_S, b. ν_I.

Supplementary Table 2: Fit parameters corresponding to fitting DEER data detected at both ν_S and ν_I using a variety of models. Data and corresponding fits are shown in F.

Model	Det. Freq.	μ / MHz	σ / MHz	V_0	λ	t_0 / μs	c_S/ μM
Exchange	ν_S	2.010	0.243	0.950	0.033	0.014	973
	ν_I	2.012	0.245	0.980	0.016	0.016	1022
Dipolar (Uniform)	ν_S	2.035	0.150	1.047	0.121	-0.042	268
	ν_I	2.038	0.150	1.028	0.061	-0.040	268
Dipolar (Weighted)	ν_S	1.947	0.133	1.027	0.108	-0.080	243
	ν_I	1.948	0.133	1.018	0.054	-0.078	249

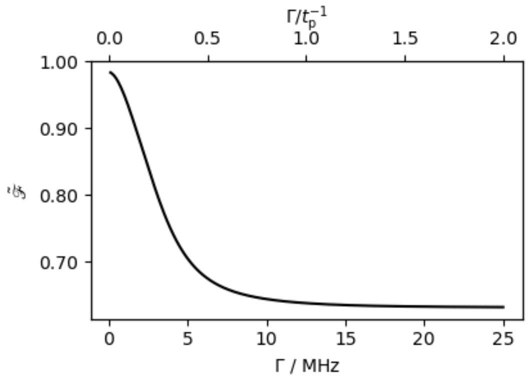

Supplementary Figure 12: Simulated pseudo-fidelity of the DFHE entangling gate as a function of spectral linewidth Γ.

Supplementary Note 4 Product operator calculations

For illustrative purposes full product operator calculations for $\hat{S}_y$ and $2\hat{S}_x\hat{I}_z$ are shown for a pump-refocus building block detecting on spin S. The results of the calculations for all operators detecting at both frequencies are shown in Supplementary Table , with detectable coherences in black and undetectable magnetisation (including detectable operators which are not refocussed into an echo) in grey. All calculations assume instantaneous, ideal pulses.

$$\alpha\hat{S}_y\hat{I}_i + \beta 2\hat{S}_x\hat{I}_z \xrightarrow{H_0 t} -\sin(\omega_\mathrm{S}t)\left[\alpha\cos\left(\frac{\omega_\mathrm{SI}t}{2}\right) + \beta\sin\left(\frac{\omega_\mathrm{SI}t}{2}\right)\right]\hat{S}_x$$

$$+\cos(\omega_\mathrm{S}t)\left[\alpha\cos\left(\frac{\omega_\mathrm{SI}t}{2}\right) + \beta\sin\left(\frac{\omega_\mathrm{SI}t}{2}\right)\right]\hat{S}_y$$

$$-\cos(\omega_\mathrm{S}t)\left[\alpha\sin\left(\frac{\omega_\mathrm{SI}t}{2}\right) - \beta\cos\left(\frac{\omega_\mathrm{SI}t}{2}\right)\right]2\hat{S}_x\hat{I}_z$$

$$-\sin(\omega_\mathrm{S}t)\left[\alpha\sin\left(\frac{\omega_\mathrm{SI}t}{2}\right) - \beta\cos\left(\frac{\omega_\mathrm{SI}t}{2}\right)\right]2\hat{S}_y\hat{I}_z$$

$$\xrightarrow{(180°)_{\hat{I}_x}} -\sin(\omega_\mathrm{S}t)\left[\alpha\cos\left(\frac{\omega_\mathrm{SI}t}{2}\right) + \beta\sin\left(\frac{\omega_\mathrm{SI}t}{2}\right)\right]\hat{S}_x + \cos(\omega_\mathrm{S}t)\left[\alpha\cos\left(\frac{\omega_\mathrm{SI}t}{2}\right) + \beta\sin\left(\frac{\omega_\mathrm{SI}t}{2}\right)\right]\hat{S}_y$$

$$+\cos(\omega_\mathrm{S}t)\left[\alpha\sin\left(\frac{\omega_\mathrm{SI}t}{2}\right) - \beta\cos\left(\frac{\omega_\mathrm{SI}t}{2}\right)\right]2\hat{S}_x\hat{I}_z$$

$$+\sin(\omega_\mathrm{S}t)\left[\alpha\sin\left(\frac{\omega_\mathrm{SI}t}{2}\right) - \beta\cos\left(\frac{\omega_\mathrm{SI}t}{2}\right)\right]2\hat{S}_y\hat{I}_z$$

$$\xrightarrow{H_0(T-t)} -\sin(\omega_\mathrm{S}T)\left[\alpha\cos\left(\frac{\omega_\mathrm{SI}(2t-T)}{2}\right) + \beta\sin\left(\frac{\omega_\mathrm{SI}(2t-T)}{2}\right)\right]\hat{S}_x$$

$$+\cos(\omega_\mathrm{S}T)\left[\alpha\cos\left(\frac{\omega_\mathrm{SI}(2t-T)}{2}\right) + \beta\sin\left(\frac{\omega_\mathrm{SI}(2t-T)}{2}\right)\right]\hat{S}_y$$

$$+\cos(\omega_\mathrm{S}T)\left[\alpha\sin\left(\frac{\omega_\mathrm{SI}(2t-T)}{2}\right) - \beta\cos\left(\frac{\omega_\mathrm{SI}(2t-T)}{2}\right)\right]2\hat{S}_x\hat{I}_z$$

$$+\sin(\omega_\mathrm{S}T)\left[\alpha\sin\left(\frac{\omega_\mathrm{SI}(2t-T)}{2}\right) - \beta\cos\left(\frac{\omega_\mathrm{SI}(2t-T)}{2}\right)\right]2\hat{S}_y\hat{I}_z$$

$$\xrightarrow{(180°)_{\hat{S}_x\hat{I}_i}} -\sin(\omega_{\mathrm{S}}T)\left[\alpha\cos\left(\frac{\omega_{\mathrm{SI}}(2t-T)}{2}\right)+\beta\sin\left(\frac{\omega_{\mathrm{SI}}(2t-T)}{2}\right)\right]\hat{S}_x$$

$$-\cos(\omega_{\mathrm{S}}T)\left[\alpha\cos\left(\frac{\omega_{\mathrm{SI}}(2t-T)}{2}\right)+\beta\sin\left(\frac{\omega_{\mathrm{SI}}(2t-T)}{2}\right)\right]\hat{S}_y$$

$$+\cos(\omega_{\mathrm{S}}T)\left[\alpha\sin\left(\frac{\omega_{\mathrm{SI}}(2t-T)}{2}\right)-\beta\cos\left(\frac{\omega_{\mathrm{SI}}(2t-T)}{2}\right)\right]2\hat{S}_x\hat{I}_z$$

$$-\sin(\omega_{\mathrm{S}}T)\left[\alpha\sin\left(\frac{\omega_{\mathrm{SI}}(2t-T)}{2}\right)-\beta\cos\left(\frac{\omega_{\mathrm{SI}}(2t-T)}{2}\right)\right]2\hat{S}_y\hat{I}_z$$

$$\xrightarrow{H_0T} -[\alpha\cos(\omega_{\mathrm{SI}}t)+\beta\sin(\omega_{\mathrm{SI}}t)]\hat{S}_y\hat{I}_i+[\alpha\sin(\omega_{\mathrm{SI}}t)-\beta\cos(\omega_{\mathrm{SI}}t)]2\hat{S}_x\hat{I}_z$$

Supplementary Table 3: Transformation of all Cartesian product operators under the pump-detect sequence with the refocussing pulse in $+x$.

Initial Magnetisation	Pump	Detect	Resultant Magnetisation
$\hat{1}$	I	S	$\hat{1}$
$\hat{I}_x$	I	S	$\cos(2\omega_\mathrm{I}(T-t))\cos(\omega_\mathrm{SI}t)\,\hat{I}_x + \sin(2\omega_\mathrm{I}(T-t))\cos(\omega_\mathrm{SI}t)\,\hat{I}_y - \sin(2\omega_\mathrm{I}(T-t))\sin(\omega_\mathrm{SI}t)\,2\hat{S}_z\hat{I}_x + \cos(2\omega_\mathrm{I}(T-t))\sin(\omega_\mathrm{SI}t)\,2\hat{S}_z\hat{I}_y$
$\hat{I}_y$	I	S	$\sin(2\omega_\mathrm{I}(T-t))\cos(\omega_\mathrm{SI}t)\,\hat{I}_x + \cos(2\omega_\mathrm{I}(T-t))\cos(\omega_\mathrm{SI}t)\,\hat{I}_y + \cos(2\omega_\mathrm{I}(T-t))\sin(\omega_\mathrm{SI}t)\,2\hat{S}_z\hat{I}_x + \sin(2\omega_\mathrm{I}(T-t))\sin(\omega_\mathrm{SI}t)\,2\hat{S}_z\hat{I}_y$
$\hat{I}_z$	I	S	$-\hat{I}_z$
$\hat{S}_x$	I	S	$\cos(\omega_\mathrm{SI}t)\,\hat{S}_x + \sin(\omega_\mathrm{SI}t)\,2\hat{S}_y\hat{I}_z$
$2\hat{S}_x\hat{I}_x$	I	S	$\cos(2\omega_\mathrm{I}(T-t))\,2\hat{S}_x\hat{I}_x + \sin(2\omega_\mathrm{I}(T-t))\,2\hat{S}_x\hat{I}_y$
$2\hat{S}_x\hat{I}_y$	I	S	$\sin(2\omega_\mathrm{I}(T-t))\,2\hat{S}_x\hat{I}_x - \cos(2\omega_\mathrm{I}(T-t))\,2\hat{S}_x\hat{I}_y$
$2\hat{S}_x\hat{I}_z$	I	S	$-\cos(\omega_\mathrm{SI}t)\,2\hat{S}_x\hat{I}_z - \sin(\omega_\mathrm{SI}t)\,\hat{S}_y$
$\hat{S}_y$	I	S	$\sin(\omega_\mathrm{SI}t)\,2\hat{S}_x\hat{I}_z - \cos(\omega_\mathrm{SI}t)\,\hat{S}_y$
$2\hat{S}_y\hat{I}_x$	I	S	$-\cos(2\omega_\mathrm{I}(T-t))\,2\hat{S}_y\hat{I}_x - \sin(2\omega_\mathrm{I}(T-t))\,2\hat{S}_y\hat{I}_y$
$2\hat{S}_y\hat{I}_y$	I	S	$-\sin(2\omega_\mathrm{I}(T-t))\,\hat{S}_y\hat{I}_x + \cos(2\omega_\mathrm{I}(T-t))\,2\hat{S}_y\hat{I}_y$
$2\hat{S}_y\hat{I}_z$	I	S	$-\sin(\omega_\mathrm{SI}t)\,\hat{S}_x + \cos(\omega_\mathrm{SI}t)\,2\hat{S}_y\hat{I}_z$
$\hat{S}_z$	I	S	$-\hat{S}_z$
$2\hat{S}_z\hat{I}_x$	I	S	$\sin(2\omega_\mathrm{I}(T-t))\sin(\omega_\mathrm{SI}t)\,\hat{I}_x + \cos(2\omega_\mathrm{I}(T-t))\sin(\omega_\mathrm{SI}t)\,\hat{I}_y - \cos(2\omega_\mathrm{I}(T-t))\cos(\omega_\mathrm{SI}t)\,2\hat{S}_z\hat{I}_x - \sin(2\omega_\mathrm{I}(T-t))\cos(\omega_\mathrm{SI}t)\,2\hat{S}_z\hat{I}_y$
$2\hat{S}_z\hat{I}_y$	I	S	$-\cos(2\omega_\mathrm{I}(T-t))\sin(\omega_\mathrm{SI}t)\,\hat{I}_x + \sin(2\omega_\mathrm{I}(T-t))\sin(\omega_\mathrm{SI}t)\,\hat{I}_y - \sin(2\omega_\mathrm{I}(T-t))\cos(\omega_\mathrm{SI}t)\,2\hat{S}_z\hat{I}_x + \cos(2\omega_\mathrm{I}(T-t))\cos(\omega_\mathrm{SI}t)\,2\hat{S}_z\hat{I}_y$
$2\hat{S}_z\hat{I}_z$	I	S	$\hat{S}_z\hat{I}_z$
$\hat{1}$	S	I	$\hat{1}$
$\hat{I}_x$	S	I	$\cos(\omega_\mathrm{SI}t)\,\hat{I}_x + \sin(\omega_\mathrm{SI}t)\,2\hat{S}_z\hat{I}_y$
$\hat{I}_y$	S	I	$-\cos(\omega_\mathrm{SI}t)\,\hat{I}_y + \sin(\omega_\mathrm{SI}t)\,2\hat{S}_z\hat{I}_x$
$\hat{I}_z$	S	I	$-\hat{I}_z$
$\hat{S}_x$	S	I	$\cos(2\omega_S(T-t))\cos(\omega_\mathrm{SI}t)\,\hat{S}_x - \sin(2\omega_S(T-t))\sin(\omega_\mathrm{SI}t)\,2\hat{S}_x\hat{I}_z + \sin(2\omega_S(T-t))\cos(\omega_\mathrm{SI}t)\,\hat{S}_y + \cos(2\omega_S(T-t))\sin(\omega_\mathrm{SI}t)\,2\hat{S}_y\hat{I}_z$
$2\hat{S}_x\hat{I}_x$	S	I	$\cos(2\omega_\mathrm{S}(T-t))\,2\hat{S}_x\hat{I}_x + \sin(2\omega_\mathrm{S}(T-t))\,2\hat{S}_x\hat{I}_y$
$2\hat{S}_x\hat{I}_y$	S	I	$-\cos(2\omega_S(T-t))\,2\hat{S}_x\hat{I}_y - \sin(2\omega_\mathrm{S}(T-t))\,2\hat{S}_y\hat{I}_y$
$2\hat{S}_x\hat{I}_z$	S	I	$\sin(2\omega_S(T-t))\sin(\omega_\mathrm{SI}t)\,\hat{S}_x - \cos(2\omega_S(T-t))\cos(\omega_\mathrm{SI}t)\,2\hat{S}_x\hat{I}_z - \cos(2\omega_S(T-t))\sin(\omega_\mathrm{SI}t)\,\hat{S}_y - \sin(2\omega_S(T-t))\cos(\omega_\mathrm{SI}t)\,2\hat{S}_y\hat{I}_z$
$2\hat{S}_y$	S	I	$\sin(2\omega_S(T-t))\cos(\omega_\mathrm{SI}t)\,\hat{S}_x + \cos(2\omega_S(T-t))\sin(\omega_\mathrm{SI}t)\,2\hat{S}_x\hat{I}_z - \cos(2\omega_S(T-t))\cos(\omega_\mathrm{SI}t)\,\hat{S}_y + \sin(2\omega_S(T-t))\sin(\omega_\mathrm{SI}t)\,2\hat{S}_y\hat{I}_z$

Supplementary Table 3 continued: Transformation of all Cartesian product operators under the pump-detect sequence with the refocussing pulse in $+x$.

Initial Magnetisation	Pump	Detect	Resultant Magnetisation
$2\hat{S}_y\hat{I}_x$	S	I	$\sin(2\omega_S(T-t))\,2\hat{S}_x\hat{I}_x - \cos(2\omega_S(T-t))\,2\hat{S}_y\hat{I}_x$
$2\hat{S}_y\hat{I}_y$	S	I	$-\sin(2\omega_S(T-t))\,2\hat{S}_x\hat{I}_y + \cos(2\omega_S(T-t))\,2\hat{S}_y\hat{I}_y$
$2\hat{S}_y\hat{I}_z$	S	I	$-\cos(2\omega_S(T-t))\sin(\omega_{SI}t)\,\hat{S}_x + \sin(2\omega_S(T-t))\cos(\omega_{SI}t)\,2\hat{S}_x\hat{I}_z - \sin(2\omega_S(T-t))\sin(\omega_{SI}t)\,\hat{S}_y + \cos(2\omega_S(T-t))\cos(\omega_{SI}t)\,2\hat{S}_y\hat{I}_z$
$\hat{S}_z$	S	I	$-\hat{S}_z$
$2\hat{S}_z\hat{I}_x$	S	I	$-\sin(\omega_{SI}t)\,\hat{I}_y - \cos(\omega_{SI}t)\,2\hat{S}_z\hat{I}_x$
$2\hat{S}_z\hat{I}_y$	S	I	$-\sin(\omega_{SI}t)\,\hat{I}_x + \cos(\omega_{SI}t)\,2\hat{S}_z\hat{I}_y$
$2\hat{S}_z\hat{I}_z$	S	I	$2\hat{S}_z\hat{I}_z$

With the refocussing pulse is in $+x$ and the detection phase is aligned with $-y$, the real and imaginary cosine and sine oscillations relate to the in- and anti-phase operators outlined in Table 2 of the manuscript.

Supplementary Table 4: Spin operators which lead to oscillations in DEERATom readout, assuming that all coherent pulses are applied along $+x$. The operators generated by an ideal DFHE entangling gate are black, with all 'undesired' operators in grey.

Detection Spin	Operation Pair	Real Cos	Imag. Cos	Real Sin	Imag. Sin
S	1_S1_I	$\hat{S}_y$	$\hat{S}_x$	$2\hat{S}_x\hat{I}_z$	$-2\hat{S}_y\hat{I}_z$
S	1_SX_I	$\hat{S}_y$	$\hat{S}_x$	$2\hat{S}_x\hat{I}_y$	$-2\hat{S}_y\hat{I}_y$
S	1_SY_I	$\hat{S}_y$	$\hat{S}_x$	$-2\hat{S}_x\hat{I}_x$	$2\hat{S}_y\hat{I}_x$
S	X_S1_I	$-\hat{S}_z$	$\hat{S}_x$	$2\hat{S}_x\hat{I}_z$	$2\hat{S}_z\hat{I}_z$
S	X_SX_I	$-\hat{S}_z$	$\hat{S}_x$	$2\hat{S}_x\hat{I}_y$	$2\hat{S}_z\hat{I}_y$
S	X_SY_I	$-\hat{S}_z$	$\hat{S}_x$	$-2\hat{S}_x\hat{I}_x$	$-2\hat{S}_z\hat{I}_x$
S	Y_S1_I	$\hat{S}_y$	$\hat{S}_z$	$2\hat{S}_z\hat{I}_z$	$-2\hat{S}_y\hat{I}_z$
S	Y_SX_I	$\hat{S}_y$	$\hat{S}_z$	$2\hat{S}_z\hat{I}_y$	$-2\hat{S}_y\hat{I}_y$
S	Y_SY_I	$\hat{S}_y$	$\hat{S}_z$	$-2\hat{S}_z\hat{I}_x$	$2\hat{S}_y\hat{I}_x$
I	1_S1_I	$\hat{I}_y$	$\hat{I}_x$	$2\hat{S}_z\hat{I}_x$	$-2\hat{S}_z\hat{I}_y$
I	1_SX_I	$-\hat{I}_z$	$\hat{I}_x$	$2\hat{S}_z\hat{I}_x$	$2\hat{S}_z\hat{I}_z$
I	1_SY_I	$\hat{I}_y$	$\hat{I}_z$	$2\hat{S}_z\hat{I}_z$	$-2\hat{S}_z\hat{I}_y$
I	X_S1_I	$\hat{I}_y$	$\hat{I}_x$	$2\hat{S}_y\hat{I}_x$	$-2\hat{S}_y\hat{I}_y$
I	X_SX_I	$-\hat{I}_z$	$\hat{I}_x$	$2\hat{S}_y\hat{I}_x$	$2\hat{S}_y\hat{I}_z$
I	X_SY_I	$\hat{I}_y$	$\hat{I}_z$	$2\hat{S}_y\hat{I}_z$	$-2\hat{S}_y\hat{I}_y$
I	Y_S1_I	$\hat{I}_y$	$\hat{I}_x$	$-2\hat{S}_x\hat{I}_x$	$2\hat{S}_x\hat{I}_y$
I	Y_SX_I	$-\hat{I}_z$	$\hat{I}_x$	$-2\hat{S}_x\hat{I}_x$	$-2\hat{S}_x\hat{I}_z$
I	Y_SY_I	$\hat{I}_y$	$\hat{I}_z$	$-2\hat{S}_x\hat{I}_z$	$2\hat{S}_x\hat{I}_y$

Supplementary Note 5 Numerical Simulations

All simulations were performed with ν_S = 9.54 GHz and ν_I = 9.69 GHz. Pulses at each frequency only affected one set of spins to avoid unwanted echo crossings. Unless otherwise stated pulses were 80 ns long; pulses were applied along $+x$; the inter-spin coupling was 2.0 MHz; and pulses were perfectly tuned. All parameters were numerically optimized to convergence. Distributions were calculated by simulating a regularly spaced grid over the required parameter and taking a weighted sum over the corresponding distribution.

In the laboratory frame, microwave pulses can be considered to be the sum of two counter-rotating fields, with frequencies $+\omega_\mathrm{mw}$ and $-\omega_\mathrm{mw}$. In the rotating frame, oscillating at frequency $+\omega_\mathrm{mw}$, the positive term becomes time independent and the counter-rotating term oscillates with frequency $-2\omega_\mathrm{mw}$. This counter-rotating component is often ignored; however its action leads to a Bloch-Siegert phase shift, akin to a “slowing” of the rotating frame during pulses. This is particularly problematic when pulsing at frequencies close to, but not on resonance with, the rotating frame.[9] This shift was not considered during simulations as it is computationally expensive to model pulses with sufficient temporal resolution. While we did observe phase shifts experimentally, these were independent of the sign of the frequency offset and so cannot be the result of the Bloch-Siegert phase shift. We assign these phase shifts to errors in pulse formation caused by the non-uniform phase response of the pulse generator as the amplitude of the pulses was increased.

Our simulations were performed such that pulses only affected the spins they were intended to affect, that is that pulses at ν_I did not affect S spins and vice versa. This avoids the observation of artefacts in the traces which it would not be possible to prevent experimentally. Further, we did not consider distortions to the phase or amplitude of the applied pulses, assuming instead that they were generated perfectly, with a uniform B_1 field was across the sample space.

Supplementary References

1. Reginsson, G. W., Kunjir, N. C., Sigurdsson S. T., & Schiemann, O. *Chem. Eur. J.* **18**, 13580-13584 (2012).
2. Fabregas Ibanez, L.& Jeschke, G. *Phys. Chem. Chem. Phys.* **22**, 1855-1868 (2020).
3. Klauder, J. R. & Anderson, P. W. *Phys. Rev.* **125**, 912-932 (1962).
4. Bedilo, A. F. & Maryasov, A. G. *J. Mag. Reson, Series A* **116**, 87-96 (1995).
5. R. Jozsa, *J. Mod. Optics* **41**, 2315-2323 (1994).
6. Stoll S. & Schweiger, A. *J. Magn. Reson*. **178**, 42-55 (2006).
7. Fabregas Ibanez, L., Jeschke, G. & Stoll, S. *Magn Reson. (Gott)* **1**, 209-224 (2020).
8. Feintuch, A. & Vega, S. *eMagRes*, 427-452 (2017).
9. Bowman, M. K. & Maryasov, A. G. *J. Mag. Reson.* **185**, 270-282 (2007).
